# A lineage-specific rapid diagnostic test (Chagas Sero *K*-SeT) identifies Brazilian *Trypanosoma cruzi* II/V/VI reservoir hosts among diverse mammalian orders

**DOI:** 10.1371/journal.pone.0227828

**Published:** 2020-01-17

**Authors:** Mairi C. W. McClean, Tapan Bhattacharyya, Pascal Mertens, Niamh Murphy, Quentin Gilleman, Yves Gustin, Nicolas Zeippen, Samanta C. C. Xavier, Ana M. Jansen, Michael A. Miles

**Affiliations:** 1 Faculty of Infectious and Tropical Diseases, London School of Hygiene and Tropical Medicine, London, United Kingdom; 2 Coris BioConcept, Gembloux, Belgium; 3 Laboratory of Trypanosomatid Biology, Oswaldo Cruz Institute, Fiocruz, Rio de Janeiro, Brazil; Universidade Federal de Minas Gerais, BRAZIL

## Abstract

*Trypanosoma cruzi*, the protozoan agent of Chagas disease in the Americas, is comprised of six genetic lineages (TcI-TcVI) and a possible seventh (TcBat, related to TcI). Identification of *T*. *cruzi* lineages infecting reservoir mammalian species is fundamental to resolving transmission cycles. However, this is hindered by the limited sensitivity and technical complexity of parasite isolation and genotyping. An alternative approach is serology using *T*. *cruzi* lineage-specific epitopes, such as those of the trypomastigote small surface antigen (TSSA). For surveillance of *T*. *cruzi* lineage infections in mammal species from diverse Brazilian regions, we apply a novel rapid diagnostic test (RDT, Chagas Sero K-SeT), which incorporates the TSSA peptide epitope specific to TcII/V/VI (TSSApep-II/V/VI) and Protein G detection of antibodies. Chagas Sero K-SeT RDT results with sera from experimentally infected mice, from tamarin primates (*Leontopithecus* spp.) and from canines (*Canis familiaris*) were concordant with corresponding TSSApep-II/V/VI ELISAs. The Chagas Sero K-Set detected TcII/V/VI infections in *Leontopithecus* spp. from the Atlantic forest (n = 46), in *C*. *familiaris* (n = 16) and *Thrichomys laurentius* (n = 2) from Caatinga biome and Chiroptera (n = 1) from Acre, Amazonia. The Chagas Sero *K*-SeT RDT is directly applicable to TcII/V/VI-specific serological surveillance of *T*. *cruzi* infection in several different mammalian Orders. It can replace ELISAs and provides efficient, point-of-sampling, low-cost detection of TcII/V/VI infections, with at least equivalent sensitivity, although some mammals may be difficult to trap, and, not unexpectedly, Chagas Sero K-SeT could not recognise feline IgG. Knowledge of sylvatic hosts of *T*. *cruzi* can be expanded, new reservoir species discovered, and the ecology of transmission cycles clarified, particularly with adaptation to further mammalian Orders.

## Introduction

The protozoan *Trypanosoma cruzi* is the etiological agent of Chagas disease, infecting 6–7 million people (https://www.who.int/en/news-room/fact-sheets/detail/chagas-disease-(american-trypanosomiasis). Vector-borne transmission occurs via contamination of mucous membranes or abraded skin with faeces of triatomine bugs. Other transmission mechanisms include oral ingestion of triatomine contaminated food, trans-placentally, and by blood/organ donation. The initial acute phase can be fatal, particularly in infants and immunosuppressed patients. Without successful chemotherapy *T*. *cruzi* infection is usually life-long; during this chronic phase around 30% of those infected progress to chagasic cardiomyopathy, some of whom develop gastrointestinal megasyndromes [1–4].

*T*. *cruzi* is zoonotic, carried by more than 100 mammal species and 40 species of triatomine insect vectors [5]. The range of infected vectors and mammalian hosts in the Americas is from the USA in the North to southern Argentina and northern Chile. The species *T*. *cruzi* is divided into the six intra-species lineages (discrete typing units, DTUs) TcI–TcVI [6], with a seventh proposed (TcBat, related to TcI) [7]. Of particular interest is greater understanding of the cryptic natural sylvatic cycles of TcII and the hybrid lineages TcV and TcVI [8], which are associated with severe chagasic cardiomyopathy and megasyndromes in the Southern Cone countries of South America, especially in Bolivia. Historically, active transmission of TcII was common in Brazilian domestic transmission cycles. However, the cases of Chagas disease in the Brazilian Amazon basin are due predominantly to TcI, and also less frequently to TcIV and TcIII. The elucidation of the sylvatic distribution of *T*. *cruzi* lineages in Brazil has been subject to extensive research [9, 10], the TcI, TcIII and TcIV lineages are widely distributed in Brazilian mammals and biomes [11], tamarins *Leontopithecus* spp. [12] and dogs [13, 14] have been implicated by isolate genotyping as natural hosts of TcII/V/VI.

Identification of infecting *T*. *cruzi* lineage(s) by direct genotyping may be biased due to sequestration in host tissues or selection in culture, potentially in a lineage-dependent manner. This may lead to incomplete interpretation of the distribution of *T*. *cruzi* lineages in mammals. Of the many commercial in-house and rapid serological tests for human and animal *T*. *cruzi* infections, none is designed to identify *T*. *cruzi* lineage(s). The mucin Trypomastigote Small Surface Antigen (TSSA), expressed on the vertebrate bloodstream trypomastigote form, is the only *T*. *cruzi* antigen so far shown to be applicable to lineage-specific serology [15, 16]. The amino acid cores of TSSA from TcI, TcIII, and TcIV each have their own lineage-specific sequence. The TcII sequence is shared with TcV and TcVI, which have a second TcV/TcVI haplotype, and are hybrids of TcII and TcIII. Antigens based on the TSSA isoform common to TcII/V/VI have been applied to serology (ELISA) of naturally infected animals of two mammalian Orders. Cimino et al [17] used an *E*. *coli*-produced recombinant protein with dog sera from northern Argentina. Kerr et al [18] used a synthetic peptide epitope, TSSApep-II/V/VI, with Brazilian primates, confirming that *Leontopithecus rosalia* (golden lion tamarin) and *Leontopithecus chrysomelas* (golden lion-headed tamarin) may act as reservoir hosts of lineages TcII/V/VI.

An obstacle to expanding this lineage-specific serology to a wider range of mammalian Orders, and thus to resolving ecological cycles and reservoir distribution, is the availability of suitable secondary species-specific antibody conjugates. An alternative approach is to utilise conjugates linked to Protein G, produced naturally by group C and G streptococci, which binds the Fc region of a range of mammalian IgGs. We recently developed a TSSApep-II/V/VI-specific lateral flow immunochromatographic rapid diagnostic test (RDT) called Chagas Sero *K*-SeT, which incorporates Protein G conjugate, and demonstrated its efficacy with sera from Bolivian Chagas disease patients [19], and with Argentine sympatric humans and dogs [20].

Here, we apply Chagas Sero *K*-SeT to sera from experimental murine infections, and to rapid serological surveillance for TcII/V/VI infections among a range of mammalian Orders and biomes in Brazil.

## Materials and methods

### Ethics approval

Production of mouse sera adhered to the European 3Rs policy of Refinement, Reduction and Replacement (99/167/EG: Council decision of 25/1/99), took place in authorised animal facilities by licensed staff in agreement with the European Directive 86/609/EEC, and with review and approvals under UK Home office regulations [Animals (Scientific Procedures) Act 1986; project licence number 70/6997 to the London School of Hygiene and Tropical Medicine]. All Brazilian samples were obtained following the guidelines of the Animal Ethics Committee (CEUA) of the Oswaldo Cruz Institute/FIOCRUZ and all procedures followed protocols approved by the FIOCRUZ Committee of Bioethics (license LW 81/12).

### Origins of animal sera

#### Experimental infections

*Mus musculus* (strain CD1) were inoculated with 10^6^ organisms from stationary phase cultures containing infective metacyclic trypomastigotes of known biological clones of *T*. *cruzi* representing the lineages TcII (MHOM/BR/00/Y), TcIII (MDAS/PY/00/Arma18) and TcIV (MAOT/BO/00/10R26). Serum was collected at approximately 10 months post-inoculation, and serology for infection was detected using *T*. *cruzi* lysate as described below in section ‘Experimental murine sera’

#### Naturally infected mammals

**Fig 1:**
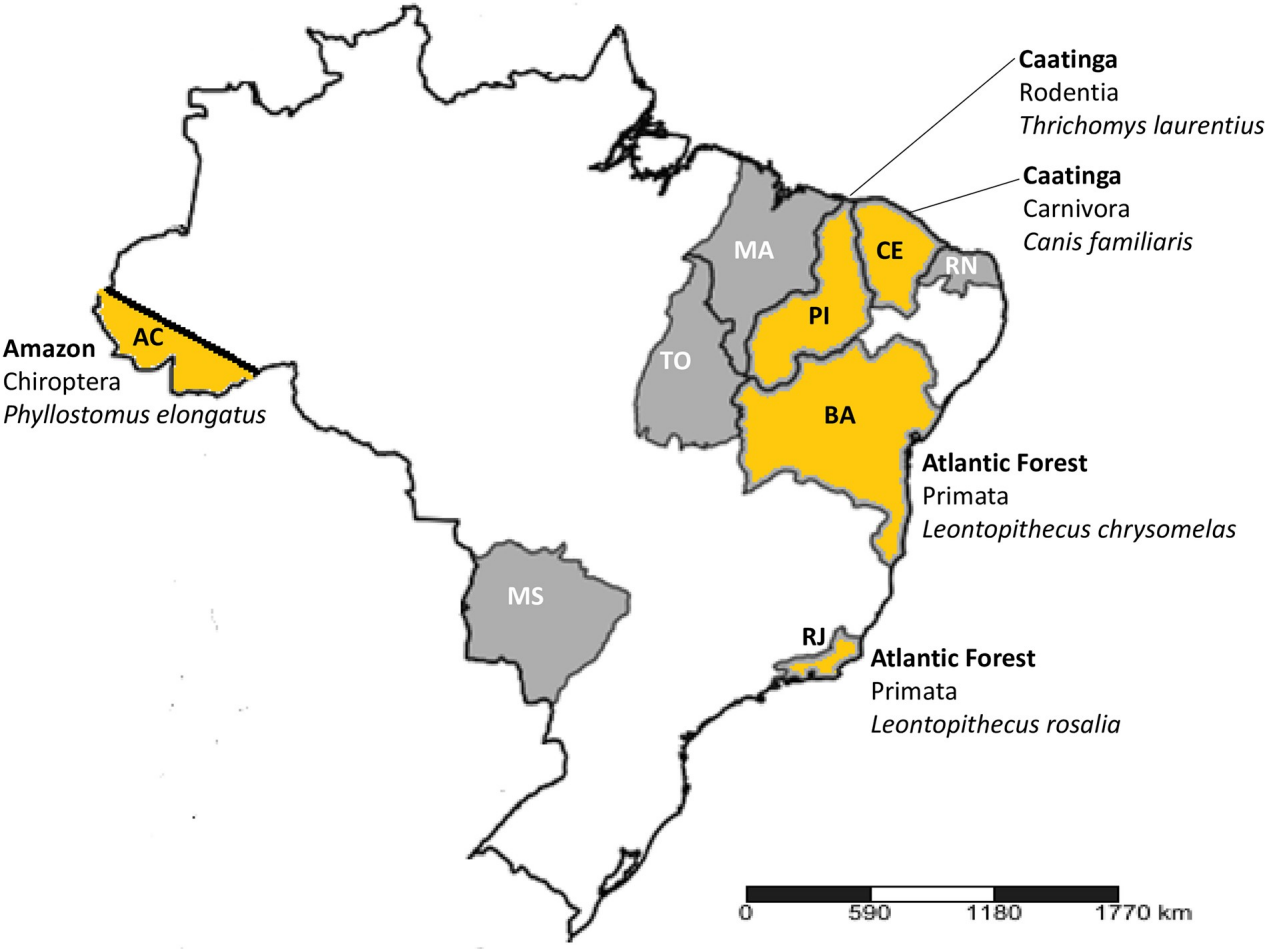
Map of Brazil showing origins of mammalian serum samples. States and biomes from which Chagas Sero *K*-SeT positive samples were identified are shown in orange; states from which no positive samples were identified are shown in grey. Abbreviations: AC, Acre; BA, Bahia; CE, Ceará; MA, Maranhão; MS, Mato Grosso do Sul; PI, Piauí; RJ, Rio de Janeiro; RN, Rio Grande do Norte; TO, Tocantins.

Sera were archived samples collected in Brazil from naturally infected mammals as part of the ongoing field research programmes of author AMJ and collaborators. Collection sites encompass a range of geographical locations, mammalian Orders, species and biomes, as shown in Fig 1, map derived from www.simplemappr.net [21].

### TSSA lineage-specific peptides

Synthetic peptides TSSApep-II/V/VI, TSSApep-III, TSSApep-IV, and TSSApep-V/VI, representing the specific TSSA epitopes of *T*. *cruzi* lineages, identified from strains described in [16], were synthesised, with N-terminal biotinylation (Table 1); details of TSSApep-II/V/VI antigenicity have been described previously [22, 23]. The indirect fluorescent antibody test (IFAT) was applied to all naturally- infected serum samples.

**Table 1:** *Trypanosoma cruzi* lineage-specific peptides (TSSApep) used in serology.

Peptide	Amino acid sequence	Representative strain	GenBank
TSSApep-II/V/VI	GTENKPATGEAPSQPG	MHOM/BR/00/Esmeraldo	GU075675
TSSApep-III	GTEKKAAAGEAPSPSG	MDAS/CO/00/CM17	GU075674
TSSApep-IV	GTDKKTAAGEAPSPSG	MHOM/BR/00/CanIII	GU075671
TSSApep-V/VI	GTENKPAAGEAPSQPG	MINF/BR/00/CL Brener	GU075678

Polymorphic residues are underlined. Peptides were N-terminal biotinylated.

### Lineage-specific TSSApep ELISA

Replica assays were performed simultaneously in duplicate plates. TSSA peptide (TSSApep) ELISAs were performed with the four lineage-specific peptides shown in Table 1. Mean values of optical density (OD) were calculated from the duplicate plates; cut-off values were mean negative serum values + 3 SD, with at least two reference negatives in every ELISA plate. Positive/negative controls for each sylvatic mammal type were used according to the results of previous serology by immunofluorescence.

#### Experimental murine sera

96-well flat bottomed plates (735–0465: Immulon 4HBX, VWR, UK) were coated with 1 μg/100 μL/well of avidin (A9275: Sigma-Adlrich, UK) diluted in coating buffer (15 mM Na_2_CO_3_, 34 mM NaHCO_3_, pH 9.6) for capture of biotinylated lineage-specific peptide (Table 1). As positive controls, separate wells were coated with lysate of *T*. *cruzi* TcII (IINF/PY/00/Chaco23) at 0.2 μg/100 μL/well. Plates were covered with an adhesive sheet (676001: Greiner Bio-one, UK) and incubated overnight at 4°C. Plates were washed x 3 with PBS/0.05% (vol/vol) Tween 20 (P7949: Sigma-Aldrich) (PBS/T) and blocked with 200 μL blocking buffer (PBS/2% skimmed milk powder, Premier International Foods, UK) for 2 h at 37°C. TSSApep at 1 μg/100 μL/well in PBS/T containing 2% skimmed milk powder (PBS/T/M) was added to the avidin-coated wells, for 1 h at 37°C. After washing (x 3), 100 μL/well of a 1:100 dilution of serum in PBS/T/M was added, for 1 h at 37°C. After washing (x 6), 100 μL/well of Peroxidase-AffiniPure Goat Anti-Mouse IgG (H + L) antibody (polyclonal; Jackson Immunoresearch, USA; Cat. no. 115-035-003, Lot no. 45266; Antibody Registry AB_10015289) diluted 1:5000 in PBS/T/M was added, for 1 h at 37°C. After washing (x 6), plates were developed with substrate comprised of 100 μL/well of 50 mM phosphate/citrate buffer (pH 5.0) containing 2 mM o-phenylenediamine HCl (OPD; P1526: Sigma-Aldrich) and 0.009% (vol/vol) H_2_O_2_ (216763: Sigma-Aldrich), and plates developed in the dark at room temperature for 10 minutes. Substrate reactions were stopped by addition of 2M H_2_SO_4_ (50 μL/well), and absorbance read at 490 nm.

#### Primate sera

ELISA plates were coated, blocked, and received TSSApep as described in Table 1 as described above. Thereafter, 100 μL/well of a 1:200 dilution of primate serum in PBS/T/M was applied, for 1 h at 37°C. Following washing (x 6), 100 μL/well of goat anti-human IgG-peroxidase (polyclonal; Sigma-Aldrich, USA; Cat. no. A0170; Antibody Registry AB_257868) diluted 1:5000 in PBS/T/M was added, for 1 h at 37°C. After washing (x 6), plates were developed with 100 μL/well of 3,3’,5,5’-Tetramethylbenzidine (TMB; Bio-Manguinhos, Fiocruz, Brazil) in the dark at room temperature. After stopping the reaction, absorbances were read at 450 nm.

#### Canine sera

ELISA plates were coated directly with 1 μg/100 μL/well TSSApep in coating buffer overnight, without avidin, because we observed that some dog sera bound non-specifically to avidin. After washing and blocking steps as described above, 100 μL/well of 1:200 dilution of canine serum in PBS/T/M was added and incubated, for 1 h at 37°C. After washing (x 6), 100 μL/well of 1:10 000 dilution of Peroxidase-AffiniPure Rabbit Anti-Dog IgG (H+L) antibody (polyclonal; Jackson Immunoresearch, USA; Cat. no. 304-035-003, Lot no. 105408; Antibody Registry AB_2339344) was added, for 1 h at 37°C. ELISA development was by Biomanghuinos TMB as described above, or alternatively by TMB ELISA substrate (ab171523: Abcam, UK). The dilutions of sera and peroxidase-conjugated antibody were previously optimised by titration.

#### Feline sera

ELISA plates were coated, blocked, and received TSSApep as described for murine sera. Thereafter, 100 μL/well of a 1:100 dilution of feline serum in PBS/T/M was applied, for 1 h at 37°C. After washing (x 6), 100 μL/well of rabbit anti-cat Ig-HRP (horse radish peroxidase, polyclonal; Sigma-Aldrich, UK; Cat. no. SAB3700080, Lot no. RI21730) diluted 1: 10 000 was added. Development was by OPD or TMB.

#### Other mammalian sera

Coating, blocking, addition of TSSApep and of 1:100 dilutions of sera were as described for canine samples. IgG was assayed with the following conjugates according to type of mammal: for armadillo, 1:1000 dilution of Protein G-HRP (P8170; Sigma-Aldrich, UK); for opossum, 1:5000 dilution of goat anti-opossum IgG (H+L)-HRP conjugate (polyclonal; Alpha Diagnostic International, USA; Cat. no. 30815-HP); for coati, 1:5000 dilution of goat anti-raccoon IgG-HRP (polyclonal; Bethyl Laboratories, USA; Cat. no. A140-123P; Antibody Register AB_1966121); for bat, 1:5000 dilution of goat-anti bat IgG (H+L)-HRP (polyclonal; Bethyl Laboratories, USA; Cat. no. A140-118P; Antibody Register AB_309475).

#### Comparison of Protein A and Protein G peroxidase conjugates

In comparative pilot assays, Protein A-HRP (Southern Biotech, USA: Cat. no. 7300–05) and Protein G- HRP (Fisher Scientific, UK; Cat. no. 11899150) were used at 1:1000 dilution in ELISA with sera from armadillos, cats, rodents and dogs. In these assays, TSSApep-II/V/VI (directly coated) and lysate were used separately as antigens. Reactions were developed with OPD.

### Chagas Sero *K*-SeT rapid diagnostic test (RDT)

**Fig 2:**
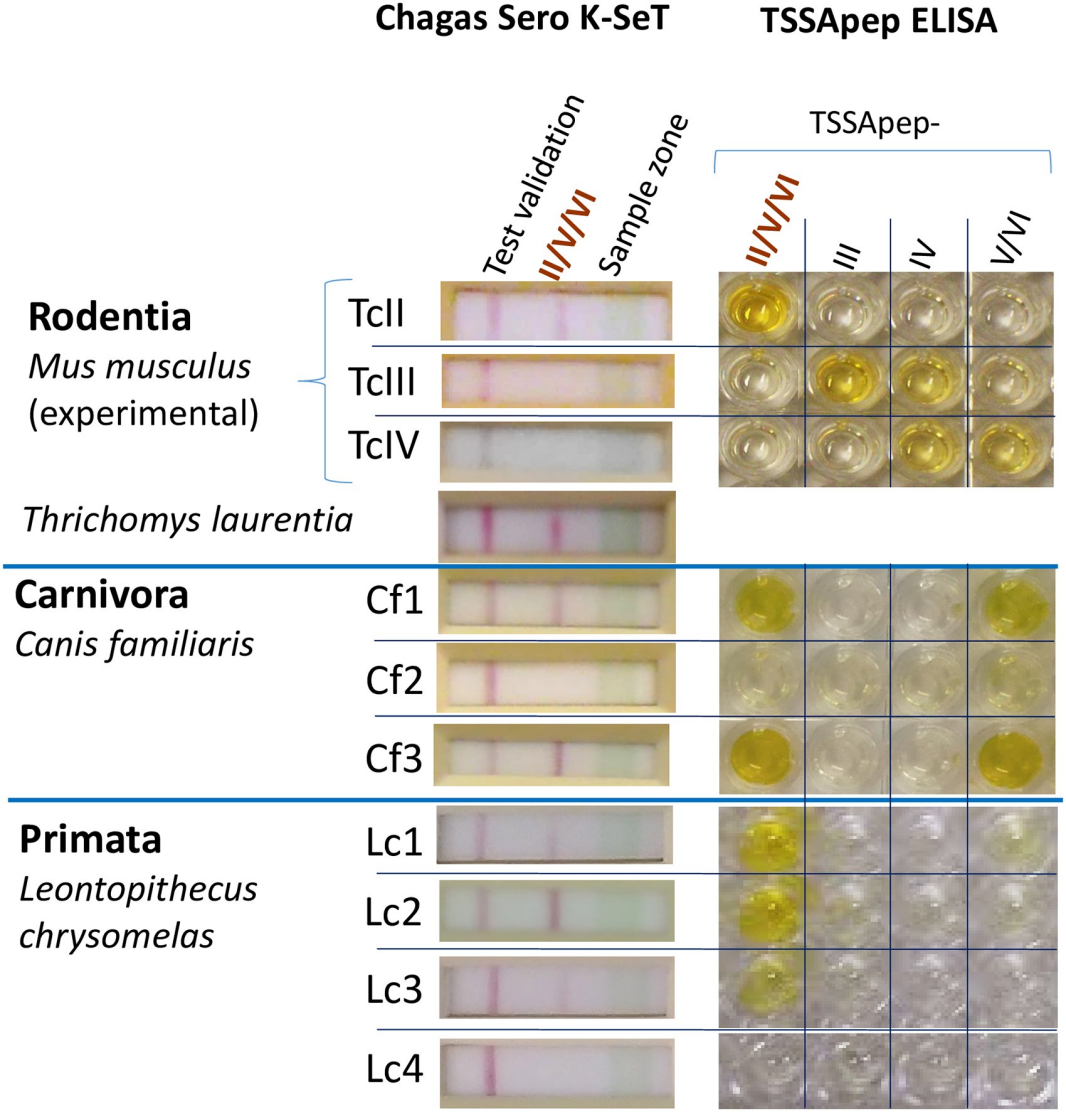
Concordance of TSSApep ELISA and Chagas Sero *K*-Set across mammalian Orders. Representative samples from experimental *T*. *cruzi* murine infections and natural infections of *Thrichomys laurentius* (Rodentia: Echimyidae), *Canis familiaris* (Carnivora: Canidae) and *Leontopithecus chrysomelas* (Primata: Callitrichidae). For primate samples, Kappa test = 0.84, 95% confidence intervals (0.64–1.00). Sample Lc4 was *T*. *cruzi* seronegative. The *T*. *laurentius* sample shown here did not have a corresponding ELISA.

This novel, low cost, lateral flow immunochromatographic rapid test was developed with Coris BioConcept, and employs TSSApep-II/V/VI as the coated antigen, and colloidal gold-labelled Protein G as the detection molecule for specific IgG, as previously described [19, 20]. Following application of the serum sample on the sample zone (wide green line, Fig 2) then buffer in the buffer window, reactions were considered positive if at 15 minutes incubation a band was observed at the antigen line, in conjunction with an integral test validation line.

### Statistical analysis

Significance of concordance between RDT results and ELISA (Kappa test) was calculated using GraphPad (GraphPad Software, San Diego, California, USA).

## Results

### Lineage-specific TSSApep ELISA

TSSApep ELISA for experimental murine, and naturally infected primate, dog and cat are described below in relation to the Chagas Sero K-SeT RDT. TSSApep ELISA data could not be obtained using the available conjugated secondary antibodies for other mammals, whether mammal-specific (bat, opossum, coati) or Protein G conjugates (armadillo).

#### Concordance of lineage-specific ELISA and Chagas Sero K-SeT RDT

Fig 2 shows representative examples of results with sera from experimental murine infections, and natural infections of rodents (*Thrichomys laurentius*), canines (*Canis familiaris*) and primates *(Leontopithecus chrysomelas*), assayed by both TSSApep ELISA and Chagas Sero K-SeT RDT.

In experimental murine infections and natural infections of Primata (Callitrichidae) and Carnivora (Canidae), TSSApep-II/V/VI ELISA and Chagas Sero *K*-SeT RDT results were concordant; *P* < 0.0001 for primates, for which most data were available (Table 2). Three of the primates TcII seropositive by ELISA were also TcV/VI ELISA positive, indicating TcV/VI infection or both TcII and TcV/VI infection. In agreement with previous ELISAs [24], serum from experimental murine infection with TcII was Chagas Sero *K*-SeT positive, whereas serum from TcIII and TcIV infections were negative by this RDT (Fig 2). Thus, not only did these two lineage-specific assays perform equally well, the colloidal gold-labelled Protein G in Chagas Sero *K*-SeT had the capacity to recognise specific IgG in diverse mammalian Orders (Rodentia, Carnivora, Primata).

**Table 2:** Chagas Sero *K*-SeT RDT serological surveillance for TcII/V/VI natural infections among Brazilian mammal species.

Order: Family	Species	Common name	Biome	Municipality, State^a^	Chagas Sero *K*-SeT positive
Primata^b^: Callitrichidae	*Leontopithecus rosalia*	Golden lion tamarin	Atlantic Forest	Silva Jardim, RJ	19/68 (27.9%)
	*Leontopithecus chrysomelas*	Golden-headed lion tamarin	Atlantic Forest	Una, BA	27/35 (77.1%)
Carnivora^c^: Canidae	*Canis familiaris*	Domestic dog	Caatinga	São Benedito, CE	16/57 (28.1%)
Chiroptera: Phyllostomidae	*Phyllostomus elongatus*	Lesser spear-nosed bat	Amazonia	Acre	1/19
Rodentia^d^: Echimyidae	*Thrichomys laurentius*	Punaré	Caatinga	PI	2/23

^a^ BA, Bahia; CE, Ceará; PI, Piaui; RJ, Rio de Janeiro.

b-d The following were all negative by Chagas Sero K-SeT:

^b^ Primata: Ten *Cebus libidinosus*, two *L*. *chrysopygus*, and a single *Alouatta belzebul*

^c^ Carnivora: Twenty nine *Nasua nasua*, nineteen *Cerdocyon thous* and four *Chrysocyon brachyurus*

^d^ Rodentia: Fourteen *Thrichomys apereoides*, five *Rattus* and single *Oligoryzomys*, *Rhipidomys*, *Nectomys*, *Akodon*.

and for the following orders not listed in the Table:

Cingulata: Forty eight *Euphractus* sp. and a single *Dasypus* sp.

Marsupialia: Eleven *Didelphis* sp. and two *Philander opossum*

(Felidae: IgG is not recognised by Protein G, see text)

### Prevalence of TcII/V/VI infections detected by Chagas Sero *K*-SeT RDT

Among the primates, the Chagas Sero *K*-SeT showed that 19 of 68 (29%) *L*. *rosalia* in Rio de Janeiro State and 27 of 35 (77%) of *L*. *chrysomelas* in the northern Brazilian State of Bahia were seropositive, indicating a high prevalence of TcII/V/VI infections, for which the natural hosts were previously poorly known. For *L*. *rosalia* and *L*. *chrysomelas*, 8 and 23 of the same samples, respectively, were previously tested by TSSApep ELISA [18]. In all but one case the Chagas Sero *K*-SeT RDT result accorded with the ELISAs; the non-matching case was positive by the RDT but negative by ELISA; Kappa test = 0.84 (95% confidence intervals: 0.64–1.00) (S1 Appendix).

Among the dogs, 16 of 57 (28%) of *C*. *familiaris* from the Caatinga biome of Ceará State were seropositive by Chagas Sero *K*-SeT.

One bat (*Phyllostomus elongatus*) of 19 examined (*Phyllostomus*, *Artibeus*, *Carollia*), and two *Thrichomys laurentius* (Fig 2) from the Caatinga biome in Piauí State were Chagas Sero *K*-SeT seropositive and thus carried a TcII and/or TcV/VI infection (Table 2).

ELISA using the anti-cat IgG secondary antibody detected two TSSApep-II/V/VI seropositive cats. Chagas Sero *K*-SeT detected no infections in felines.

Of other mammals, for which no corresponding ELISA data were available, Chagas Sero *K*-SeT found no TcII/V/VI infections in: coati (*Nasua nasua*, 29), crab-eating fox (*Cerdocyon thous*, 19), maned wolf (*Chrysocyon brachyurus*, 4); opossum (*Didelphis* and *Philander*, 13), or armadillo whether *Euphractus* (48) or *Dasypus* sp. (1) (Table 2).

The Protein G used in Chagas Sero *K*-SeT did recognise nearly a quarter or the dogs tested by this RDT; the Protein G-HRP used in ELISA failed to recognise dog IgG. The Protein A- HRP accorded with the result with specific anti-cat secondary antibody, and also recognised anti-*T*. *cruzi* lysate IgG in two armadillos.

## Discussion

Recent publications have reviewed the complexities relating to Chagas disease and *T*. *cruzi* molecular epidemiology [3, 25]. Regarding the latter point, the application of serology based on lineage-specific antigens has great potential for resolving the cryptic ecological cycles and the discovery of novel reservoir hosts of this parasite. Applications of *T*. *cruzi* lineage–specific serology to naturally-infected animals are with dogs [17, 26], primates [18] and sympatric dogs, cats, and armadillo [20]. We have applied Chagas Sero *K*-SeT to exploit the capacity of Protein G to recognise TSSApep-II/V/VI specific IgG from a range of mammalian Orders. Previous reports of RDTs for *T*. *cruzi* serology of animal reservoirs have used InBios *Trypanosoma cruzi*-Detect-Canine [27] or Chagas StatPak [28], with no lineage-specific diagnosis. We demonstrate the versatility of the Chagas Sero K-SeT to recognise TSSApep-II/V/VI specific IgG in experimental murine *T*. *cruzi* infections, and in natural infections across several Orders of Brazilian mammals, namely Primata, Carnivora (canine), Rodentia and Chiroptera.

The Chagas Sero *K*-SeT with *Leontopithecus* spp. sera demonstrated the capacity of this RDT for lineage-specific serology of primates, previously achieved only with ELISA using conjugated anti-human IgG [18]. Previous reports [29, 30] hypothesised that primates could be reservoir hosts of TcII; however, searches for TcII infections in Brazilian Amazonian primates only yielded TcI and TcIV. Our results confirm the high prevalence of TcII/V/VI infections in Brazilian *Leontopithecus* primates in the Atlantic Forest region of eastern Brazil, not in the Amazonian forest [29, 30]. Beyond Brazil, TcII was recently isolated by xenodiagnosis of a free-living capuchin (*Sapajus cay*) from eastern Paraguay [31], and *T*. *cruzi* genotypes compatible with TcII, TcV and TcVI were reported in howler monkeys (*Alouatta caraya*) in northern Argentina [32]. The occurrence of these lineages in non-contiguous areas of South America and their broad range of host genera suggest that the primate ecological cycles of these lineages are far from fully elucidated.

Rocha et al [13] genotyped TcII from Brazilian dogs, as single or mixed TcI-TcII infections in Minas Gerais State, southern Brazil. Previous reports [17, 26] have used a recombinant *E*. *coli*-produced TSSA fusion protein from CL Brener strain (TcVI; a clone from parental strain CL) in ELISA with naturally infected dogs from northern Argentina; however, the recombinant included sequences that are shared with other lineages. Chagas Sero *K*-SeT identified TcII/V/VI infections in dogs from Ceará state, in north-eastern Brazil, a region previously known to be highly endemic for TcII in domestic transmission cycles, although intradomiciliary transmission of *T*. *cruzi* has now been controlled. However, Lima et al [14] also reported TcII/V/VI and TcV/VI respectively in two dogs from Pará state, north of the Amazon Basin.

Domestic cats, which can acquire infection by eating triatomines or infected mice, are hosts of *T*. *cruzi*, as known from the early discovery of Chagas disease [33, 34], and Rocha et al [13] reported TcI from a wild ocelot (*Leopardus pardalis*) in Minas Gerais State. The two feline samples TSSApep-II/V/VI seropositive by ELISA were negative by Chagas Sero K-SeT as expected, because Protein G does not bind feline IgG [35]. We found here that Protein A could recognise feline IgG, and its further application for serological surveillance is warranted.

Regarding rodents, we show that the TSSApep-II/V/VI ELISA with anti-mouse IgG can be replaced by Chagas Sero *K*-SeT with Protein G. *Thrichomys laurentius* is a sylvatic host of *T*. *cruzi* [36, 37] in Ceará state, northeast Brazil. The TSSApep-II/V/VI seropositives identified here by Chagas Sero K-SeT were from the neighbouring state of Piauí. Similarly, a single bat specimen was positive by Chagas Sero K-Set; *T*. *cruzi* lineages have been reported from Brazilian bats, by genotyping [38, 39].

Armadillos, especially *D*. *novemcinctus*, have been reported as natural hosts of TcIII throughout South America [11, 40, 41], thus the absence of TcII/V/VI infections from all 49 armadillos was not surprising. Although Chagas Sero K-SeT was shown to be able to detect infection in armadillo [20], positive test line intensity was weak; Protein A may be more appropriate than Protein G for binding of armadillo IgG, as others have reported [42], and which we observed here. No armadillo-specific conjugate was available for ELISA; this also encourages further deployment of Protein A for these important reservoirs hosts. Opossums have been identified as common hosts of TcI but rarely reported as hosts of TcII [11, 43].

Diagnosis of *T*. *cruzi* infection has not hitherto been lineage-specific. The search for a TcI-specific antigen applicable to animals and humans remains an important current research goal, and may elucidate the reported differences in serological and immune responses in regions endemic for different lineages [44–46].

As recently described [9, 10], the association of *T*. *cruzi* lineages with hosts, biomes or habitats is complex and not fixed. Infecting lineage composition may fluctuate within mammalian populations over time if there is varying exposure and transmission efficiency. Mixed infections, such as TcI and TcII, may occur in single mammals, and are not currently detectable by lineage-specific serology; nevertheless, due to sustained infection antibodies to TcII should not be lost. The Chagas Sero K-SeT RDT described here does not make redundant *T*. *cruzi* genotyping, or advanced analytical techniques such as flow cytometry [47]; however, by detecting lineage-specific host IgG, it can provide epidemiological information when direct parasite genotyping is hampered or subject to confounding biases.

## Conclusion

The biological and geographical range of mammals from which specific anti-TSSApep-II/V/VI IgG has been identified by Chagas Sero *K*-SeT RDT demonstrates its great potential in identifying novel reservoir hosts and elucidating ecological cycles of lineage transmission. We have shown here that this rapid, easy to use and interpret, Protein G-based RDT can replace ELISA in *T*. *cruzi* TSSApep-II/V/VI lineage-specific serology of a range of mammalian Orders; it does not require isolation, culture or direct genotyping of parasites, nor is it subject to confounders of those approaches. The combined use of Protein A and Protein G (or Protein A/G) as detection molecules for IgG may increase further the range of mammalian Orders to which lineage-specific serology can be applied.

## Supporting information

S1 Appendix Primate data used for Kappa analysis. (XLSX)
