# Correction: A lineage-specific rapid diagnostic test (Chagas Sero *K*-SeT) identifies Brazilian *Trypanosoma cruzi* II/V/VI reservoir hosts among diverse mammalian orders

**DOI:** 10.1371/journal.pone.0231566

**Published:** 2020-04-02

**Authors:** Mairi C. W. McClean, Tapan Bhattacharyya, Pascal Mertens, Niamh Murphy, Quentin Gilleman, Yves Gustin, Nicolas Zeippen, Samanta C. C. Xavier, Ana M. Jansen, Michael A. Miles

In Fig 2, the depiction of the ELISA results of the experimental murine serum is in the incorrect orientation in relation to the rest of the figure. The authors have provided a corrected version here.

**Fig 2:**
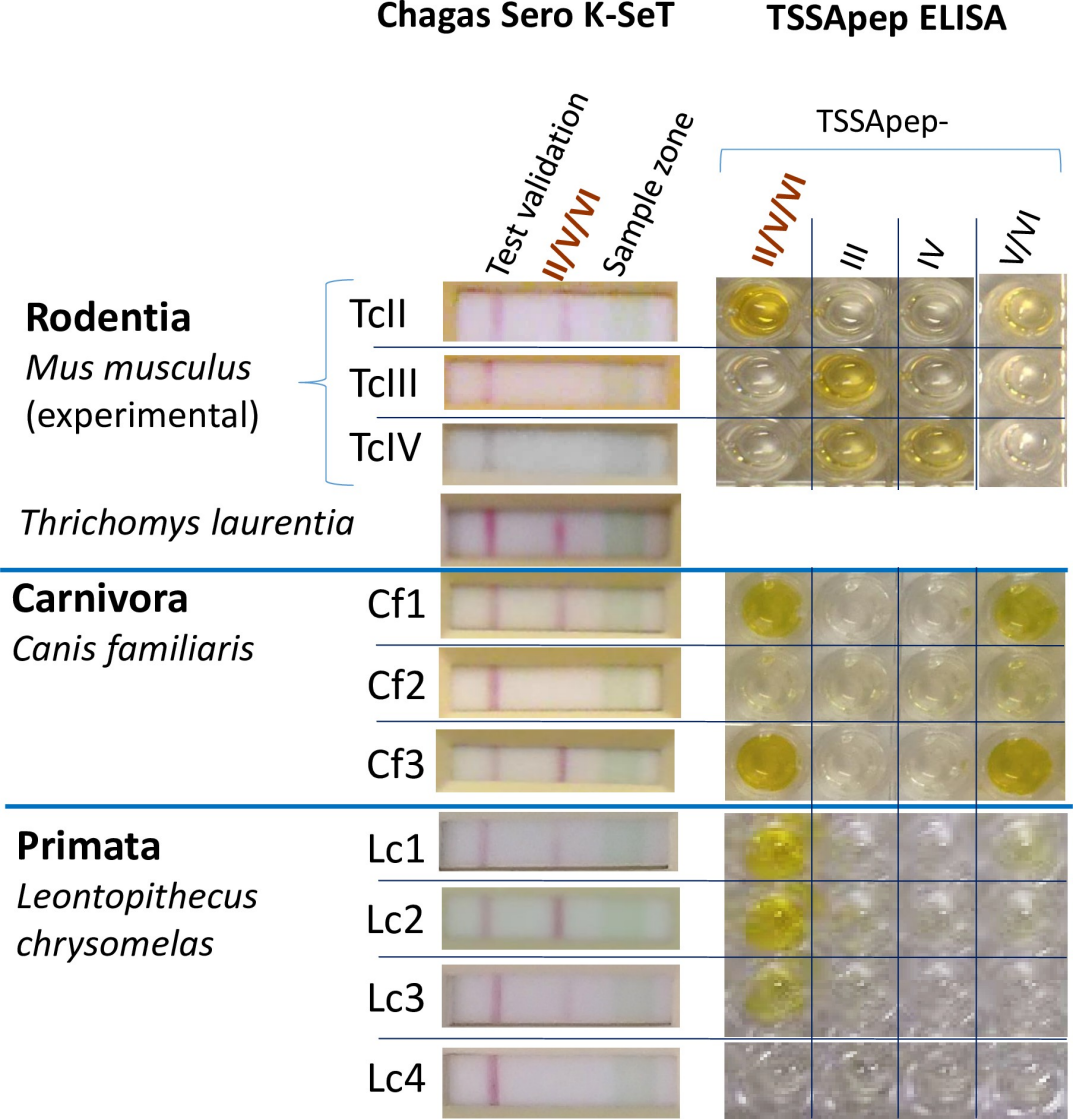
Concordance of TSSApep ELISA and Chagas Sero *K*-Set across mammalian Orders. Representative samples from experimental *T*. *cruzi* murine infections and natural infections of *Thrichomys laurentius* (Rodentia: Echimyidae), *Canis familiaris* (Carnivora: Canidae) and *Leontopithecus chrysomelas* (Primata: Callitrichidae). For primate samples, Kappa test = 0.84, 95% confidence intervals (0.64–1.00). Sample Lc4 was *T*. *cruzi* seronegative. The *T*. *laurentius* sample shown here did not have a corresponding ELISA.
